# Refractory cardiopulmonary failure after glyphosate surfactant intoxication: a case report

**DOI:** 10.1186/1745-6673-4-2

**Published:** 2009-01-30

**Authors:** Chirn-Bin Chang, Chia-Chu Chang

**Affiliations:** 1 Nephrology Division, Department of Internal Medicine, Changhua Christian Hospital, Changhua, Taiwan; 2 College of Health Sciences, Institute of Medical Research, Chang Jung Christian University, Tainan, Taiwan

## Abstract

**Background:**

Glyphosate is an herbicide considered to be of low toxicity to humans because its effects are specific to plants. However, fatal reactions to glyphosate have been reported after the ingestion of large amounts. Pulmonary edema, shock, and arrhythmia were the reported causes of mortality.

**Case presentation:**

We present the case of a 57-year-old woman who was admitted to the emergency department unconsciousness after ingestion of glyphosate surfactant in a suicide attempt. Metabolic acidosis, refractory respiratory failure, and shock developed during hospitalization. Despite aggressive supportive care, the patient died in the hospital.

**Conclusion:**

The toxicokinetics of glyphosate surfactant is complicated. Respiratory failure, metabolic acidosis, tachycardia, elevated creatinine, and hyperkalemia are poor prognostic factors if presented. Physicians should consider using hemodialysis early to improve the outcome of patients with glyphosate surfactant intoxication.

## Background

Several fatal cases of glyphosate surfactant intoxication were reported in the literature from 1991 to 2008 [1-8]. The toxicokinetics of glyphosate in humans has not been well established because of its complicated toxicity [9]. Early prognostic factors were analyzed and reported to classify patients with severe intoxication [5,8]. No antidotal therapy is available; therefore, hemodialysis has been used to treat patients with glyphosate surfactant intoxication, whose symptoms included arrhythmia, shock, hyperkalemia, and metabolic acidosis, despite supportive care [6,10]. This case is presented to increase awareness of the symptoms experienced by patients with severe glyphosate surfactant intoxication. Early intensive care is necessary, and the necessity of hemodialysis should be determined for these patients [6,10].

## Case presentation

### Our case

**Figure 1:**
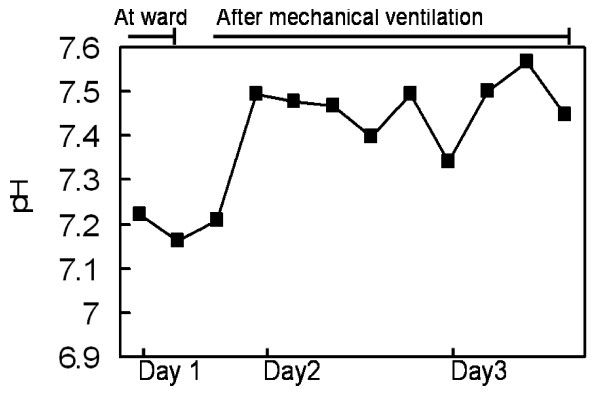
**Serial levels of serum pH**.

**Figure 2:**
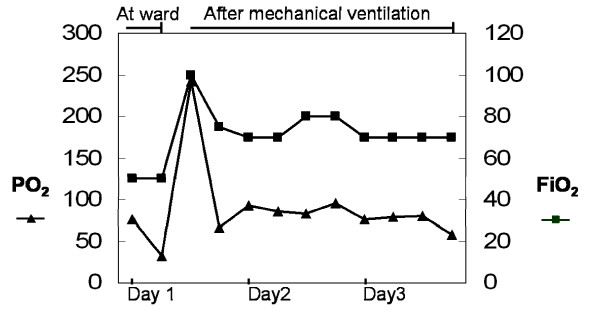
**PaO2 and FiO2 levels during hospitalization**.

**Figure 3:**
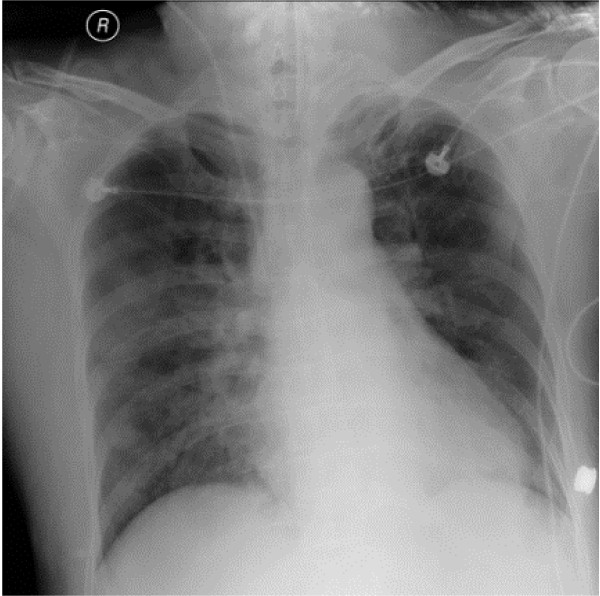
**Infiltration over bilateral lung fields**.

A 57-year-old woman with a medical history of Grave's disease and breast infiltrating ductal carcinoma, which was treated with a radical mastectomy, was brought to the emergency department by ambulance within 50 minutes after unconsciousness found by family. She had attempted suicide by ingesting estimated 400 ml of nian-nian-chun (Chinese brand name for glyphosate surfactant) but her family did not known the exact time of ingestion. This product contains 41% glyphosate isopropylamine and 15% polyoxyethyleneamine. On admission, the patient was in a state of drowsy consciousness, had a Glasgow Coma Scale (GCS) of E1V1M2, and was diaphoretic, incontinent, and vomiting. Her vital signs on admission to the emergency department were as follows: blood pressure, 120/70 mm Hg; pulse, 87 beats/min; respiration, 22 breaths/min; and temperature, 35°C. Physical examination showed an injected throat, oral ulcers, blood-tinged saliva, crackles on chest auscultation, cold extremities, and unremarkable findings concerning the head, neck, heart, genital organs, and rectum. Serum benzodiazepine, alcohol and organophosphate and urinary paraquet concentrations were examined to excluded other drugs related unconsciousness. The results of laboratory studies at emergency department were as follows: non-fasting plasma glucose, 156 mg/dL; creatinine, 1.2 mg/dL; leukocytes, 11600/μl; segmented neutrophils, 74.4%; and potassium, 5.6 mEq/L. Gastric irrigation was performed after ingestion for one hour, but no obvious herbicide was detected. The patient regained consciousness (GCS: E3V5M6) in 30 minutes at emergency department, but shock and respiratory failure developed in 5 hours after admission to the hospital. A blood gas analysis before endotracheal intubation showed mixed metabolic and respiratory acidosis (pH: 7.223; PCO_2_: 30.8 mm Hg; HCO_3_: 12.8 mmol/L; BE: – 12.9 mmol/L). She was transferred to the intensive care unit, mechanically ventilated, and treated according to the critical care principle. The hyperkalemia was corrected with insulin/glucose infusion and kayexalate ingestion. The serum level of potassium decreased to 3.4 mEq/L on the second day of admission. The acidemia was corrected by intermittent sodium bicarbonate infusion (Figure 1). However, refractory shock persisted despite the administration of fluids, dopamine, vasopressin, epinephrine, and norepinephrine. A low ratio of FiO_2 _to PaO_2 _(Figure 2) and bilateral lung infiltration (Figure 3) developed. Non-sustained ventricular tachycardia developed on the third day of admission. Amiodarone was loaded with 150 mg. There was no more ventricular arrhythmia but pulseless electric activity was noted. Cardiopulmonary resuscitation was performed for 30 minutes but there was no spontaneous pulse. The patient died 3 days after being admitted to the hospital.

## Discussion

The toxicokinetics of glyphosate alone in humans is not well established, and most of what is known has been derived from animal studies. On ingestion, glyphosate is initially distributed to the small intestine, colon, kidney, and bone; the majority is rapidly excreted without biotransformation in the urine [9].

There are different formulations of surfactant. The product names and the chemical constituents were as Agri-Dex^®^- (Polyol fatty acid esters, Polyoxyethyl polyol fatty acid esters and Paraffin base petroleum oil), LI-700^®^- (Phosphatidylcholine, Propionic acid, and Alkylpolyoxyethylene ether), R-11^®^- (Octylphenoxypolyethoxyethanol, n-Butanol and Compounded silicone), Latron AG-98^®^-AG- (Octylphenoxypolyethoxyethanol, isopropanol and Polydimethylsiloxane), and Latron AG-98^®^-N- (Nonylphenoxypolyethoxyethanol, n-Buthanol and silicone antifoam compound). Other surfactant was used including polyethoxylaed alyl etheramine, trimethylethoxypolyoxypropylammonium chloride, polyethoxysorbitan monolaurate and alkyl polysaccharide.

In one case report, the post-mortem examination of tissue samples analyzed by HPLC/PCR confirmed concentrations of 100 ppm in the brain, 550 ppm in the blood, 60 ppm in the liver, and 3650 ppm in the kidney [2]. The pattern of absorption, metabolism, and elimination was similar in animal studies [9]. In our patient, the transient consciousness maybe related to transient high concentration in brain. Then, the plasma declined rapidly in 2 hours after ingestion, therefore; her consciousness regained.

Respiratory distress, shock, metabolic acidosis, and hyperkalemia are all predictors of poor outcome [5,8]. Round-up pneumonitis, aspiration pneumonia, and pulmonary edema are all possible causes of respiratory distress. Increased acid production (which affects ATP consumption and production), metabolic derangements (which cause increased acid production and impair the renal elimination of acids) are all possible causes of metabolic acidosis [11]. Shock may be related to primary cardiovascular effects or to secondary effects from acidosis or electrolyte imbalance. The cardiovascular effects of glyphosate surfactant were examined in the aorta and heart of rat. Vasorelaxation and inhibition of heart twitch tension were observed in the study by Chan et al [12]. Bradycardia and ventricular arrhythmia often develop and are fatal in humans [4,7]. It may also have primary toxicity in the conduction system and secondary toxicity in the circulation system. In our patient, electrolyte imbalance was corrected and acidemia was improved with sodium bicarbonate treatment; however, refractory shock persisted. The cardiovascular effect of glyphosate surfactant can be complicated. Some clinicians treat toxin-induced metabolic acidosis with a buffer, such as sodium bicarbonate, to correct acidemia. However, it has not been definitively shown to improve mortality in patients with metabolic acidosis after the administration of sodium bicarbonate [11].

Three studies of glyphosate surfactant intoxication in Taiwan have been published. In one of these studies (published in 1991), 7 of 93 patients died after exposure to glyphosate surfactant. The authors concluded that being older than 40 years and having a large ingestion volume are risk factors for mortality [1]. In another of these studies (published in 2000), 11 of 131 patients died within 2.8 days of exposure. The authors identified three risk factors (pulmonary edema, acidosis, and hyperkalemia) associated with a poor prognosis [5]. In the last of these studies (published in 2008), 17 of 58 patients died from glyphosate surfactant intoxication. The authors found 5 factors present at emergency department to be associated with mortality: respiratory failure, metabolic acidosis, tachycardia, elevated creatinine, and hyperkalemia [8]. In our patient, there were respiratory failure, metabolic acidosis, elevated creatinine, pulmonary edema and hyperkalemia initially related to poor prognosis.

Although certain symptoms and signs indicate the severity of poisoning [1], the three abovementioned studies identified specific factors associated a high risk of mortality in patients with glyphosate surfactant intoxication [1,5,8]. Physicians can use these factors to classify the mortality risk of these patients at the time of admission to the emergency department. Those patients with a high risk of mortality should be admitted to the intensive care unit immediately, and their cardiovascular activity should be closely monitored. Four patients reportedly presented with shock, acidosis, or hyperkalemia refractory to aggressive supportive treatment after glyphosate surfactant intoxication [2,6,10]. Their condition improved dramatically after hemodialysis was initiated to correct acidosis, hyperkalemia, or an unstable hemodynamic status. However, five other patients reportedly died despite hemodialysis [5,7]. Indication of emergent dialysis was suggested in previous article [10] according to poor prognostic factors and the suggestion of Acute Dialysis Quality Initiative (ADQI) workgroup. These are listed in Table 1. The effects of dialysis were improvement of hemodynamic status and correction of electrolyte imbalance related to renal failure. It was also suspected that dialysis could eliminate the active metabolite of glyphosate surfactant [6].

**Table 1:** Indication of emergent dialysis

Indication of emergent dialysis
Large volume ingestion (> 200 ml)
Urine output < 0.5 ml/kg/h
Serum creatinine > 1.5 or GFR decreased by 25%
Volume overload- unresponsive to diuretics
Respiratory compromise- including pulmonary edema and hypoxia
Cardiovascular dysfunction- shock or EKG abnormalities
Electrolyte changes- hyperkalemia and acidemia

## Conclusion

The toxicokinetic of glyphosate surfactant is complicated and the further detailed study is necessary to reveal definite mechanism of human toxicity. There are poor prognostic factors after analyze patients' presentation after intoxication. Physicians should consider using hemodialysis early to improve the outcome of patients with severe glyphosate surfactant intoxication.

## Abbreviations

GCS: Glasgow Coma Scale; HPLC: high performance liquid chromatograpy; PCR: polymerase chain reaction; ATP: adenosine triphosphate; ppm: parts per million.

## Consent

Written informed consent was obtained from the patient for publication of this case report.

## Competing interests

The authors declare that they have no competing interests.

## Authors' contributions

CBC contributed in visiting the case, all authors contributed in editing the manuscript, all authors contributed in drafting the manuscript, all authors read and approved the final manuscript.
